# Clinical Efficacy and Nephrotoxicity of the Loading Dose Colistin for the Treatment of Carbapenem-Resistant *Acinetobacter baumannii* in Critically Ill Patients

**DOI:** 10.3390/pharmaceutics14010031

**Published:** 2021-12-24

**Authors:** Wasan Katip, Suriyon Uitrakul, Peninnah Oberdorfer

**Affiliations:** 1 Department of Pharmaceutical Care, Faculty of Pharmacy, Chiang Mai University, Chiang Mai 50200, Thailand; 2 Epidemiology Research Group of Infectious Disease (ERGID), Chiang Mai University, Chiang Mai 50200, Thailand; aoberdor@med.cmu.ac.th; 3 Department of Pharmaceutical Care, School of Pharmacy, Walailak University, Nakhon Si Thammarat 80160, Thailand; Suriyon.ui@wu.ac.th; 4 Department of Pediatrics, Division of Infectious Diseases, Faculty of Medicine, Chiang Mai University, Chiang Mai 50200, Thailand

**Keywords:** loading dose, colistin, critically ill patients, CRAB, efficacy, safety

## Abstract

Carbapenem-resistant *Acinetobacter baumannii* (CRAB) is one of the most common causes of nosocomial infections in critically ill patients. Colistin methanesulfonate (CMS), an inactive prodrug, has been considered as a last-resort treatment for CRAB infection in critically ill patients. The objective of this study was to assess 30-day survival and nephrotoxicity in critically ill patients who received non-loading dose (LD) versus LD of CMS for CRAB infection treatment. Between 2012 and 2017, this retrospective cohort analysis was performed at Chiang Mai University Hospital (CMUH), focusing on critically ill patients with CRAB infection who received either non-LD or LD of CMS. A total of 383 patients met the criteria for inclusion. At the 30th day of treatment, the survival rate of patients in the LD CMS group was 1.70 times (adjusted HR) of those in the non-LD group (95% CI = 1.17–2.50, *p* = 0.006). Clinical response was significantly higher in the LD CMS group than non-LD CMS group (aHR, 1.35, 95% CI, 1.01–1.82, *p* = 0.046). In addition, a microbiological response—eradication of pre-treatment isolated pathogens in post-treatment cultures—in patients with LD CMS was 1.57 times that of patients with non-LD CMS (95% CI, 1.15–2.15, *p* = 0.004). Additionally, there was a significant difference in nephrotoxicity between LD CMS and non-LD CMS (aHR, 1.57, 95% CI, 1.14–2.17, *p* = 0.006). Based on these results, LD CMS should be used to increase the opportunity of patients to achieve favourable outcomes. However, LD CMS was found associated with an increase in nephrotoxicity, so renal function should be closely monitored when LD colistin was administered.

## 1. Introduction

In critically ill patients, carbapenem-resistant *Acinetobacter baumannii* (CRAB) has been established as the main treatment for difficult-to-treat nosocomial infection [1]. The prevalence of CRAB has increased globally over several decades [2]. Outbreaks of CRAB have been observed in a number of hospitals, especially in intensive care units (ICU) [3,4,5,6]. Although *Acinetobacter baumannii* was originally considered a low virulent pathogen [7,8], it has recently been well documented that patients infected with CRAB are at a high risk of mortality [9,10].

Colistin is an antibacterial agent that is made out of polymyxins, specifically polymyxin E. It has good efficacy against Gram-negative bacteria, including Pseudomonas aeruginosa and *Acinetobacter baumannii*, as well as CRAB [11]. Previously, this drug was used very little due to its nephrotoxicity side effect, but recently it has subsequently returned in favour as a last-line of defence against carbapenem-resistant Gram-negative bacteria [11]. The recommended route of administration for colistin is intermittently intravenous (IV) infusion. Colistin methanesulfonate (CMS) is an inactive prodrug of colistin that has shown gradual CMS conversion and then caused slow rising of colistin concentrations in plasma. Therefore, when CMS is given to patients, particularly those who are critically ill, the loading dose (LD) of CMS is highly recommended to rapidly increase the serum concentration of the drug [11].

Based on the estimation of unbound colistin concentrations and the previously constructed semi-mechanistic pharmacokinetic and pharmacodynamics (PK/PD) model explaining bacterial elimination, loading dosages of colistin base activity (CBA) higher than 66.66 to 100 mg (1 to 3 million units [MU]) demonstrated a remarkable increase in initial bacterial killing. Moreover, pharmacokinetic studies recommended a loading dose of 200 to 300 mg CBA (6 to 9 MU) in critically ill patients; such administration significantly provided faster bacterial eradication and higher clinical cure rate [12]. Plasma concentrations of produced colistin were reported to increase slowly over several hours or even days after the start of CMS therapy in critically ill patients [13]. Based on many PK/PD studies, the 2019 international consensus guidelines on the optimal use of polymyxins recommend starting IV therapy with a 300 mg CBA (9 MU) of CMS loading dose administered over 0.5–1 h, followed by the first maintenance dose within 12–24 h [13]. This guideline also recommends some possible requirements for future research, such as safety and efficacy of loading doses [13]. Regarding the recommended issue, the clinical studies relating to efficacy and safety outcomes of LD CMS in critically ill patients are still limited. As a result, the objective of this study was to assess the efficacy and safety of high LD of CMS compared with non-LD of CMS in treatment of CRAB infection in critically ill patients.

## 2. Materials and Methods

This was a retrospective cohort study that was conducted at Chiang Mai University Hospital (CMUH). This tertiary teaching hospital consists of 1400 beds that annually receives approximately 1,300,000 outpatients and 48,000 inpatients. The study was per-formed between January 2012 and August 2017. The ethical committee for human research at Chiang Mai University’s Faculty of Medicine approved a waiver of informed consent for this study which retrospectively collected and anonymously presented the data. All procedures were carried out in conformity with the applicable rules and regulations. Patients with monomicrobial infection of carbapenem-resistant and colistin-sensitive *A. baumannii* were included. The microbiological database and patient medication records were reviewed in order to identify patients with CRAB infections. The US Centres for Disease Control and Prevention (CDC) [14] and infectious disease physicians’ evaluations were utilized to identify and classify the infections. Patients were included if they were assessed to be critically ill by physicians, older than or equal to 18 years, admitted to the intensive care unit (ICU), and had received CMS for more than 2 days to treat a confirmed CRAB infection. Only patients who had never received any other treatment with CRAB eradication activities were included in the study. Patients with colonisers or contaminants in the CRAB cultures, as well as those with incomplete records, were excluded. The patients were separated into two groups: those who received a high LD and those who did not receive a LD. Patients in the LD CMS group were administered a LD of 300 mg CBA, followed by a maintenance dose of 150 mg CBA every 12 h, whilst the other group were administered only 150 mg of CBA every 12 h.

### 2.1. Outcome Measurement

The 30-day survival rate, clinical response, and bacteriological response to the treatment were stated to determine efficacy. The primary outcome of this study was a 30-day survival rate, which was defined as patient survival at the 30th day after starting medication. The secondary outcomes of interest included clinical response and microbiological response. Clinical response to treatment was measured by resolution or partial resolution of presenting symptoms and signs of CRAB infection at the completion of CMS treatment. Failure to fulfil all criteria for clinical response during CMS treatment was defined as clinical failure. Microbiological response at the end of therapy was defined as the absence of CRAB in two consecutive cultures of patient samples that were obtained from the site of infection after the initial positive culture, whereas microbiological failure was defined as the presence of CRAB in subsequent specimen cultures. Clinical symptoms as well as laboratory findings were used to assess safety of the patients. The RIFLE criteria [15] together with the judgment of physicians were used to define colistin-induced nephrotoxicity. If patients presented any grade of renal dysfunction based on the RIFLE criteria [15], nephrotoxicity was counted.

### 2.2. Antimicrobial Susceptibility Testing

All causal bacteria were identified using standard microbiological techniques. The standard disc diffusion method and an automated bacterial identification and susceptibility testing system (VITEK 2 system, bioMérieux, Marcy I ‘Etoile, France) were used to test susceptibility of the organisms. The Clinical and Laboratory Standards Institute (CLSI) method [16] was used to assess antimicrobial susceptibility. The VITEK 2 system was used to test *A. baumannii* antibiotic susceptibility, and broth microdilution was used to test susceptibility to colistin, with resistance defined as a colistin minimum inhibitory concentration (MIC) breakpoint more than 2 µg/mL. The VITEK 2 system is a fully automated system that uses fluorogenic technology to identify organisms and a turbidimetric method to measure susceptibility [17]. *A. baumannii* was classified as CRAB if it was resistant to any carbapenem but susceptible to colistin.

### 2.3. Statistical Analysis

To compare the differences in the main results, descriptive statistics were used to analyse categorical data including percentage, frequency, average, and standard deviation in order to explain the general features and fundamental information of the patients. Fisher’s exact test was used to compare baseline categorical data. Other statistical approaches included independent T-test for normally distributed data and Mann–Whitney U test for non-normally distributed data. The significance threshold of all analyses was 0.05.

Propensity score inverse probability weighted (IPW) regression was used to adjust potential biases due to imbalanced baseline characteristics of the treatment and control groups. Using multivariable logistic regression, the propensity score of covariates including hypertension, cardiovascular disease, diabetes mellitus, chronic kidney disease, chronic obstructive pulmonary disease, septic shock, mechanical ventilation during infection, Charlson score, APACHE II score, baseline serum creatinine, baseline glomerular filtration rate (GFR), total CMS dose, vasopressor, diuretic drug, pneumonia, and urinary tract infection were obtained and used adjustment with an inclusion criterion of *p* < 0.25.

The weights were then used to evaluate outcomes for two groups using cause-specific Cox proportional hazards regression models to account for the time to event. The primary and secondary outcomes were investigated using a univariate Cox regression analysis. A Cox proportional hazards regression model was used to estimate the adjusted hazard ratios (HR) and 95 percent confidence intervals (CI) of relevant components (IPW using the propensity score for baseline covariate adjustment). For all analyses, a 2-sided *p*-value of 0.05 was considered statistically significant. 

Covariates with *p*-value ≤ 0.25 in the univariate nephrotoxicity analysis and other variables (i.e., age, gender, hypertension, cardiovascular disease, diabetes mellitus, chronic kidney disease, chronic obstructive pulmonary disease, malignancy, mechanical ventilation, Charlson score, APACHE II score, baseline SCr, baseline GFR, total CMS dose, aminoglycosides, diuretics, amphotericin B, vasopressor and vancomycin) that showed the trend toward association with nephrotoxicity were entered into the Cox regression multivariable model in a backward stepwise elimination to identify independent predictors of nephrotoxicity in all critically ill patients. Finally, the complete model eliminated one factor at a time until all factors remained statistically significant at a 5% significance level, regardless of their *p*-values. The adjusted Hazards ratio (aHR) and 95 percent confidence interval were used to express the results of the Cox regression (CI). Stata software, version 14 was used to analyse the data (Stata Corp, College Station, TX, USA).

## 3. Results

There were 383 critically ill patients with CRAB infections who met the inclusion criteria and recruited for analysis. Of them, 239 cases (62.40%) were female, and the average age of all patients was 66.15 ± 16.08 years. Hypertension and coronary artery disease were the most common underlying diseases (Table 1). Two hundred and fifty-nine patients (67.62%) were in the LD CMS group and 124 patients (32.38%) were in the non-LD group. The comparisons of patient characteristics between LD and non-LD groups are shown in Table 1.

The median minimum inhibitory concentrations (MICs) of colistin against CRAB in the LD CMS group and non-LD CMS group were 0.25 µg/mL. All CRAB isolates in this study were susceptible to colistin.

The results of the univariate analysis of 30 days survival rate was calculated (156/383, 40.73%), with 30-day survival rates of 42.08% and 37.90% of the patients in the LD and non-LD CMS groups (Crude HR: 1.35, 95% CI: 0.96 to 1.91; *p* = 0.082), respectively. The total rate of clinical response was 54.83% (210/383), divided into 54.83% in the LD CMS and 54.84% in the non-LD CMS groups (Crude HR: 1.21, 95% CI: 0.91 to 1.62; *p* = 0.189). The average rate of microbiological response was 56.65% (217/383), with microbiological response rates of 57.91% and 54.03% in patients with and without LD CMS therapy (Crude HR: 1.30, 95% CI: 0.97 to 1.73; *p* = 0.073), respectively. Additionally, the rate of nephrotoxicity at any stage of RIFLE criteria (risk, injury, failure, loss of kidney function, and end-stage kidney disease) was 56.76% and 32.26% in the LD and non-LD CMS groups (Crude HR: 2.01, 95% CI: 1.47 to 2.85; *p* = 0.001), respectively. All analyses of crude outcomes after univariate analysis are shown in Table 2.

**Figure 1:**
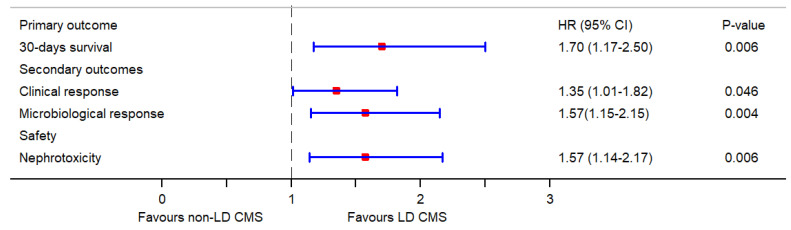
Forest plot of hazard ratios and 95% confidence intervals of outcomes for critically ill patients receiving LD CMS and non-LD CMS therapy.

The multivariate Cox regression analysis (IPW using the propensity score for baseline covariate adjustment) showed that LD CMS was associated with a significant increase in 30-day survival rate (aHR: 1.70, 95% CI: 1.17–2.50; *p* = 0.006), clinical response (aHR: 1.35, 95% CI: 1.01–1.82; *p* = 0.046) and microbiological response (aHR: 1.57, 95% CI: 1.15–2.15; *p* = 0.004) as compared to non-LD CMS. However, a LD CMS substantially increased the risk of nephrotoxicity (aHR: 1.57, 95% CI: 1.14–2.17; *p* = 0.006), compared to non-LD CMS (Table 2). All outcomes for critically ill patients receiving LD CMS and non-LD CMS therapy are shown in Figure 1.

### Risk Factors Associated with Nephrotoxicity

We found that the significant risk factors for nephrotoxicity were elderly (age ≥ 60 years; aHR: 2.06, 95% CI: 1.96 to 2.17; *p* = 0.001), received vasopressors (aHR: 1.22, 95% CI: 1.11 to 1.34; *p* = 0.001), received LD CMS (aHR: 1.70, 95% CI: 1.07 to 2.70; *p* = 0.026), male (aHR: 1.45, 95% CI: 1.29 to 1.63; *p* = 0.001), received amphotericin B (aHR: 1.08, 95% CI: 1.02 to 1.16; *p* = 0.016), and high APACHE II score (aHR: 1.03, 95% CI: 1.01 to 1.04; *p* = 0.001) (Table 3).

## 4. Discussion

The efficacy of LD CMS versus non-LD CMS in the treatment of critically ill patients with CRAB infections was investigated in this study. Our findings showed that patients treated with LD CMS had greater 30-day survival rate, higher clinical response and higher microbiological responses than patients treated with non-LD CMS. However, the incidence of nephrotoxicity was also higher in patients with LD CMS than non-LD CMS. Based on these findings, to achieve favourable outcomes, LD CMS is suggested together with close monitoring of renal function.

The labelling dose instructions on the package of CMS might not always result in optimal therapeutic serum concentrations. Several pharmacokinetic studies have shown that a CMS dose higher than currently indicated on package labels might provide significant clinical benefits. Many recent studies have also shed light on the pharmacodynamic (PD) and pharmacokinetic (PK) aspects of colistin. The colistin’s PD profile is summarized as concentration dependent [18,19,20], so the ratio of area under the curve to the MIC (AUC/MIC ratio) is the best PK-PD parameter to reflect the effectiveness profile of colistin, particularly if the therapeutic goal is 50 to 65 mg h/L [18,21,22].

The difference in chemical composition (degree of methanesulfonation) of the CMS among a different brand or batch of available CMS products in the market is suspected relating to the concentration of colistin in patient serum [13]. For a brand or batch that slowly converts to colistin, the justification for a loading dose would be more persuasive. Unfortunately, there is currently no method to predict the rate of in vivo conversion for a specific batch. Additionally, the mechanism of loading dose colistin that affects the risk of developing kidney injury is still unclear [13]. However, in case patients need urgent antibiotic delivery, the therapeutic benefits of a loading dose may outweigh the risk of nephrotoxicity [13]. Although the guidelines recommend the initiation IV CMS therapy with a loading dose of 300 mg CBA (9 MU), this recommendation is based on many studied PK/PD data. Therefore, there is still a need for more information on the safety and efficacy of the loading dose colistin [13].

Dosing recommendations based on novel pharmacokinetic data, i.e., loading dose, were presented by Garonziket al. [21] and are now being used in clinical practice. As a result, some studies have tried to investigate the advantage of this LD regimen. For instance, Dalfino et al. [23] revealed favourable results in their cohort of 28 patients with bloodstream infections and ventilator-associated pneumonias. The patients receiving high-dose, extended-interval CMS were reported to achieve an 82.1% clinical cure rate [23]. However, there was no control group to compare the results in the mentioned study. Additionally, only small numbers of patient were studied. Another prospective observational study [24] was conducted to investigate the pharmacokinetics of CMS and colistin in critically ill patients, as well as potential relationship to clinical effectiveness and renal function. Twenty critically ill adult patients with colistin-susceptible multidrug-resistant (MDR) infections and normal renal function were treated with 9 MU LD of CMS, followed by a maintenance dose of 3 MU three times a day for 24 h. Klebsiella pneumoniae and *Acinetobacter* spp. were two major pathogens of infection in those patients. The results indicated that their clinical cure rate was 50% (10/20). Average of peak concentrations of colistin post-loading dose in the “cure” and “failure” groups were 3.0 ± 1.1 mg/L (1.75–5.14) and 2.37 ± 1.2 mg/L (1.52–5.54), respectively (*p* = 0.13). On the seventh day of treatment, the rate of nephrotoxicity was 5% [24]. 

This recent study, with more recruited patients, observed a significantly higher 30-day survival rate, higher clinical response and higher microbiological response in patients utilising LD CMS group as compared to non-LD CMS group. These findings were consistent with many prior studies [25,26,27]. For example, Trifi et al. [25] compared high-dose CMS to regular dosage CMS in 92 patients with multidrug-resistant, Gram-negative bacilli infection. The results showed that patients with high-dose CMS had a greater clinical cure rate than those with regular doses (63% versus 41.3 %, *p* = 0.04) [25]. However, this study did not include critically ill patients to be analysed.

Another retrospective, single-centre, cohort study [26] was performed with 127 patients; 45 (35%) patients received a high-dose of CMS (9 MU per day, 300 mg CBA), whereas 82 (65%) patients received a low-dose of the drug. Using bivariate and multivariate analyses, a high dose CMS was found to be correlated with 7-day global cure (40% versus 19.5%; *p* = 0.013) with an odds ratio (OR) of 3.40; 95%CI, 1.37 to 8.45; *p* = 0.008). Nonetheless, this study did not report the incidence of acute renal injury due to the drug [26].

Similarly, a retrospective study [27] evaluated the target average steady-state total plasma concentrations (Css,avg) of colistin that affected the efficacy and safety of IV CMS therapy in critically ill patients. A total of 153 critically ill patients (71% were men) who received IV CMS were included for analysis. The desired goal of Css,avg of CMS was calculated using each patient’s daily CMS dose and creatinine clearance. There was no significant indicator of clinical cure found in this study. However, based on multiple logistic regression analysis, the use of IV CMS loading dose was significantly associated with microbiological eradication (OR: 2.783, 95% CI: 1.126–6.880; *p* = 0.027). Colistin-induced nephrotoxicity also significantly less appeared in individuals who received inhaled colistin compared to systemic CMS therapy (OR: 0.331, 95% CI: 0.119–0.925; *p* = 0.035) [27]. 

There is a meta-analysis [28] that aimed to evaluate the effect of LD together with high-dose maintenance regimens of CMS on the rate of treatment success and risk of nephrotoxicity. A total of 1115 patients from eight studies (three prospective and five retrospective studies) were analysed. The results indicated that LD CMS associated with higher microbiological success [risk ratio (RR) = 1.23, 95% CI = 1.10–1.39] but not clinical success (RR = 1.04, 95%CI = 0.87–1.24). Additionally, no significant relationships were observed for nephrotoxicity (RR = 1.31, 95% CI = 0.90–1.91) and mortality (RR = 1.03, 95% CI = 0.82–1.29). In addition, this meta-analysis suggested that future studies are needed to determine the dose schedule that will present the best balance between therapeutic efficacy and safety [28].

Although previous studies [25,26,27] reported positive outcomes among patients receiving high doses of CMS for the treatment of various infections caused by Gram-negative bacteria, they did not aim to compare LD-CMS and non-LD CMS treatment in critically ill patients [25,26,27].

Nephrotoxicity is one of the most frequent and serious side effects in patients treated with colistin. Several previously published studies discovered and reported that nephrotoxicity—evaluated by the RIFLE criteria—ranged from 20% to 69% with a dose-dependent effect [29,30,31]. In the present study, nephrotoxicity was observed in 56.76% of the patients in LD CMS group and 32.26% of the patients in non-LD dose group. This rate was consistent with several earlier studies as well as our previous studies [29,30,31,32,33]. Additionally, those studies indicated that colistin-induced nephrotoxicity appeared to be dose dependent [29,30,31,32,33]. Likewise, the results in this recent study showed that LD CMS was independently associated with nephrotoxicity (HR: 1.57, 95% CI: 1.14–2.17; *p* = 0.006). Moreover, multivariate Cox regression analysis was used to identify variables that were independently associated with nephrotoxicity in patients. It was found that vasopressor use, amphotericin B use, LD CMS administration, male gender, high APACHE II score, and older than 60 years of age were associated with an increased risk of nephrotoxicity. Therefore, close monitoring of the renal function is needed in critically ill patients with at least one of these risk factors.

Regarding the fact that a 9 MU LD of CMS is not always administered in real clinical practice, there is a possibility that many patients usually receive lack of LD or inadequate LD CMS (less than 9 MU). According to the study of Giacobbe et al. [34] that assessed CMS use in an area with common multidrug-resistant Gram-negative bacteria, the results showed that 79% of patients (221 adult patients) received a LD of 9 MU of CMS and 85% of them received sufficient maintenance doses [34]. Focusing Chiang Mai hospital, there are some clinicians who do not follow the international guideline recommendation of 9 MU LD of CMS. However, one of the inclusion criteria of this study was only patients who received 300 mg CBA (equivalent to 9 MU) were categorised as LD CMS. It was therefore assured that all patients of the LD group in this study received an adequate loading dose of CMS. Additionally, the observed results in this study should be applied to only patients receiving 9 MU LD of CMS and should not be extrapolated to other doses of loading dose colistin.

There were several limitations in the present study. Firstly, significant variation in patient characteristics between the therapy groups was still observed, despite the fact that the authors tried to adjust them with various methods. In fact, the detected differences were also found in other retrospective investigations and were arduous to eliminate. A propensity score IPW method was applied to manage the known baseline characteristics. Furthermore, Cox proportional hazards regression was applied to adjust variables that were different between the LD CMS and non-LD CMS groups. Secondly, because this was a single-centre study, the distribution of MIC might have varied depending on local epidemiology, and potentially affected the impact of outcomes. Thirdly, the current study was limited to a certain age group. The average age of patients in this study was 65.71 years and 66.35 years in the LD and non-LD CMS groups, respectively. This highlighted that most patients were old and therefore more susceptible to develop drug resistance than a younger population, due to a weak immune system and other clinical history. The application of findings in this study should focus on elderly rather than critically ill patients at all ages.

Generally, the present study is a part of the vast literature related to the use of CMS, and demonstrably considers the serious issues of LD CMS in severe patients suffering from CRAB infections. This study also reiterates the well-known nephrotoxic effects associated with the LD CMS, which have always led to an absolutely cautious use of the CMS. In addition, the data obtained by the present study also confirm the need to monitor LD CMS therapy. The present study should therefore be considered as a further confirmation of what is already known on the clinical use of colistin, particularly in Thai hospitals.

## 5. Conclusions

When comparing non-LD to LD CMS, this study found a strong correlation between LD CMS and several outcomes in critically ill patients with CRAB infections, including 30-day survival, and clinical and microbiological rates. However, LD CMS was also significantly correlated with the incidence of nephrotoxicity. Therefore, based on the results, LD CMS should be used in critically ill patients with CRAB infection, but rigorous and close monitoring of the renal function is also needed. In addition, nephrotoxicity should be more extensively monitored and appropriately managed if patients are older than 60 years, are male, use vasopressors, use amphotericin B, or have high APACHE II scores.

## Figures and Tables

**Table 1:** Demographic and clinical characteristics of patients received LD CMS compared to non-LD CMS therapy.

Characteristic	Non-LD CMS (*n* = 124)	LD CMS (*n* = 259)	*p*-Value
Sex, *n* (%)			
Male	50 (40.32)	94 (36.29)	0.499
Female	74 (59.68)	165 (63.71)	
Age, mean ± SD (year)	65.71 ± 16.19	66.35 ± 16.06	0.717
Duration of treatment ± SD (days)	9.95 ± 6.22	8.91 ± 7.00	0.161
Comorbidities * *n* (%)	109 (87.90)	234(90.35)	0.478
Hypertension	66 (53.23)	108(41.70)	0.038
Cardiovascular disease	51 (41.13)	76(29.46)	0.028
Diabetes mellitus	37 (29.84)	38 (14.67)	0.001
Chronic kidney disease	56 (45.16)	36(13.95)	0.001
Chronic obstructive pulmonary disease	16(12.90)	50(19.31)	0.148
Malignancy	26 (20.97)	70 (27.03)	0.211
Chronic liver disease	8(6.45)	17(6.59)	1.000
Septic shock, *n* (%)	120 (96.77)	242 (93.44)	0.233
Mechanical ventilation, *n* (%)	115 (92.74)	223 (86.10)	0.063
Charlson Score, mean ± SD	4.88 ± 2.41	4.42 ± 2.20	0.066
APACHE II score, mean ± SD	13.57± 4.03	12.69 ± 4.22	0.054
Baseline SCr, mg/dl, median (IQR)	1.6 (0.9–2.7)	1.0(0.5–1.9)	0.003
Baseline GFR, ml/min, median (IQR)	38.75 (16.5–65.09)	65.45 (29.08–100.47)	0.001
Total CMS dose, mean ± SD (g)	1.61 ± 1.38	2.19 ± 1.49	0.001
Type of nephrotoxic medications ^#^, *n* (%)			
Aminoglycosides	4 (3.23)	6 (2.32)	0.733
Diuretics	91 (73.39)	224 (86.49)	0.003
Amphotericin B	14 (11.29)	24 (9.27)	0.585
Vasopressor	120 (96.77)	242 (93.44)	0.233
Vancomycin	88 (70.97)	167 (64.48)	0.247
Length of hospital stay, mean ± SD (day)	39.33 ± 26.69	37.66 ± 28.51	0.582
Site of CRAB infection			
Pneumonia	108 (87.10)	210 (81.08)	0.149
Bacteremia	4 (3.23)	14 (5.41)	0.444
UTI	11 (8.87)	36 (13.90)	0.185
Other	5 (4.03)	15 (5.79)	0.625
Colistin MICs, µg/mL, median (min-max)	0.25 (0.084–1.5)	0.25 (0.064–1.5)	0.868

SCr, serum creatinine; SD, standard deviation; UTI, urinary tract infection; Other, inter costal drainage and surgical site infection; IQR, interquartile range; MIC, minimum inhibitory concentration; * Each patient could have more than 1 diseases; ^#^ Each patient could have more than 1 drugs.

**Table 2:** Cox regression analysis of outcomes for critically ill patients receiving LD CMS and non-LD CMS therapy (*n* = 383).

Variable	Non-LD CMS (*n* = 124)	LD CMS (*n* = 259)	Crude HR (95% CI)	*p*-Value	Adjusted HR * (95% CI)	*p*-Value
**Efficacy** **Primary outcome**						
30 days survival	47 (37.90)	109 (42.08)	1.35 (0.96–1.91)	0.082	1.70 (1.17–2.50)	0.006
**Secondary** **outcomes**						
Clinical response	68 (54.84)	143 (54.83)	1.21 (0.91–1.62)	0.189	1.35 (1.01–1.82)	0.046
Microbiological response	67 (54.03)	150 (57.91)	1.30 (0.97–1.73)	0.073	1.57(1.15–2.15)	0.004
**Safety**						
Nephrotoxicity (RIFLE criteria)	40 (32.26)	147 (56.76)	2.01 (1.47–2.85)	0.001	1.57 (1.14–2.17)	0.006
- Risk	19 (15.32)	44 (16.98)				
- Injury	9 (7.25)	46 (17.76)				
- Failure	10 (8.06)	56 (21.62)				
- Loss	1 (0.80)	1 (0.38)				
- ESRD	1(0.80)	0 (0.00)				

CI, confidence interval; * Inverse probability weighting using the propensity score for baseline covariate adjustment; HR, hazard ratio.

**Table 3:** Multivariable Cox regression model for significant predictors of risk factors for nephrotoxicity among all critically ill patients.

Variable ^a^	Non-Nephrotoxicity (*n* = 188)	Nephrotoxicity (*n* = 187)	aHR (95% CI)	*p* Value
LD CMS	112 (59.57)	147 (78.61)	1.70 (1.07–2.70)	0.026
Age ≥ 60	118 (62.77)	141 (75.40)	2.06 (1.96–2.17)	0.001
Male	63 (33.51)	77 (41.18)	1.45 (1.29–1.63)	0.001
Vasopressor	173 (92.02)	181 (96.79)	1.22 (1.11–1.34)	0.001
Amphotericin B	16 (8.51)	21 (11.23)	1.08 (1.02–1.16)	0.016
APACHE II score	14 (13–19)	19 (15–22)	1.03 (1.01–1.04)	0.001
Baseline GFR	52.14 ± 25.66	46.20 ± 26.06	1.00 (1.00–1.01)	0.144

^a^ Factors that were evaluated but did not remain in the stepwise backward regression model included baseline serum creatinine, hypertension, cardiovascular disease, diabetes mellitus, chronic kidney disease, chronic obstructive pulmonary disease, malignancy, mechanical ventilation, Charlson score, total CMS dose, aminoglycosides, diuretics and vancomycin.

## Data Availability

The datasets used and analysed during the current study are available from the corresponding author on reasonable request.

## References

[1] Munoz-Price L.S., Weinstein R.A. (2008). *Acinetobacter* infection. N. Engl. J. Med..

[2] Peleg A.Y., Seifert H., Paterson D.L. (2008). *Acinetobacter baumannii*: Emergence of a Successful Pathogen. Clin. Microbiol. Rev..

[3] Kohlenberg A., Brümmer S., Higgins P., Sohr D., Piening B.C., De Grahl C., Halle E., Rüden H., Seifert H. (2009). Outbreak of carbapenem-resistant *Acinetobacter baumannii* carrying the carbapenemase OXA-23 in a German university medical centre. J. Med. Microbiol..

[4] Garlantézec R., Bourigault C., Boles J., Prat G., Baron R., Tonnelier J., Cosse M., Lefèvre M., Jourdain S., Lelay G. (2011). Cost-analysis of an intensive care unit closure due to an imipenem-resistant oxa-23 *Acinetobacter baumannii* outbreak. J. Hosp. Infect..

[5] Ayraud-Thévenot S., Huart C., Mimoz O., Taouqi M., Laland C., Bousseau A., Castel O. (2012). Control of multi-drug-resistant *Acinetobacter baumannii* outbreaks in an intensive care unit: Feasibility and economic impact of rapid unit closure. J. Hosp. Infect..

[6] Molter G., Seifert H., Mandraka F., Kasper G., Weidmann B., Hornei B., Öhler M., Schwimmbeck P.G., Kröschel P., Higgins P. (2016). Outbreak of carbapenem-resistant *Acinetobacter baumannii* in the intensive care unit: A multi-level strategic management approach. J. Hosp. Infect..

[7] Blot S., Vandewoude K., Colardyn F. (2003). Nosocomial bacteremia involving *Acinetobacter baumannii* in critically ill patients: A matched cohort study. Intensiv. Care Med..

[8] Garnacho J., Sole-Violan J., Sa-Borges M., Diaz E., Rello J. (2003). Clinical impact of pneumonia caused by *Acinetobacter baumannii* in intubated patients: A matched cohort study. Crit. Care Med..

[9] Falagas M.E., Kopterides P., Siempos I.I. (2006). Attributable mortality of *Acinetobacter baumannii* infection among critically ill patients. Clin. Infect. Dis..

[10] Jones C.L., Clancy M., Honnold C., Singh S., Snesrud E., Onmus-Leone F., Mc Gann P., Ong A.C., Kwak Y., Waterman P. (2015). Fatal Outbreak of an Emerging Clone of Extensively Drug-Resistant *Acinetobacter baumannii* With Enhanced Virulence. Clin. Infect. Dis..

[11] Grégoire N., Mimoz O., Mégarbane B., Comets E., Chatelier D., Lasocki S., Gauzit R., Balayn D., Gobin P., Marchand S. (2014). New Colistin population pharmacokinetic data in critically Ill patients suggesting an alternative loading dose rationale. Antimicrob. Agents Chemother..

[12] Mohamed A.F., Karaiskos I., Plachouras D., Karvanen M., Pontikis K., Jansson B., Papadomichelakis E., Antoniadou A., Giamarellou H., Armaganidis A. (2012). Application of a Loading Dose of Colistin Methanesulfonate in Critically Ill Patients: Population Pharmacokinetics, Protein Binding, and Prediction of Bacterial Kill. Antimicrob. Agents Chemother..

[13] Tsuji B.T., Pogue J.M., Zavascki A.P., Paul M., Daikos G.L., Forrest A., Giacobbe D.R., Viscoli C., Giamarellou H., Karaiskos I. (2019). International Consensus Guidelines for the Optimal Use of the Polymyxins: Endorsed by the American College of Clinical Pharmacy (ACCP), European Society of Clinical Microbiology and Infectious Diseases (ESCMID), Infectious Diseases Society of America (IDSA), International Society for Anti-infective Pharmacology (ISAP), Society of Critical Care Medicine (SCCM), and Society of Infectious Diseases Pharmacists (SIDP). Pharmacotherapy.

[14] Horan T.C., Andrus M., Dudeck M.A. (2008). CDC/NHSN surveillance definition of health care–associated infection and criteria for specific types of infections in the acute care setting. Am. J. Infect. Control.

[15] Ricci Z., Cruz D., Ronco C. (2008). The RIFLE criteria and mortality in acute kidney injury: A systematic review. Kidney Int..

[16] Clinical and Laboratory Standards Institute (2010). Performance Standards for Antimicrobial Susceptibility Testing: Twentieth Informational Supplement M100-S20.

[17] Ece G., Samlioglu P., Atalay S., Kose S. (2014). Evaluation of the in vitro colistin susceptibility of *Pseudomonas aeruginosa* and *Acinetobacter baumannii* strains at a tertiary care centre in Western Turkey. Infez. Med..

[18] Bergen P.J., Bulitta J.B., Forrest A., Tsuji B.T., Li J., Nation R.L. (2010). Pharmacokinetic/Pharmacodynamic Investigation of Colistin against *Pseudomonas aeruginosa* Using an In Vitro Model. Antimicrob. Agents Chemother..

[19] Owen R.J., Li J., Nation R.L., Spelman D. (2007). In vitro pharmacodynamics of colistin against *Acinetobacter baumannii* clinical isolates. J. Antimicrob. Chemother..

[20] Zhou Y.-F., Liu P., Zhang C.-J., Liao X.-P., Sun J., Liu Y.-H. (2020). Colistin combined with tigecycline: A promising alternative strategy to combat escherichia coli harboring blaNDM–5 and mcr-1. Front. Microbiol..

[21] Garonzik S.M., Li J., Thamlikitkul V., Paterson D., Shoham S., Jacob J., Silveira F.P., Forrest A., Nation R.L. (2011). Population Pharmacokinetics of Colistin Methanesulfonate and Formed Colistin in Critically Ill Patients from a Multicenter Study Provide Dosing Suggestions for Various Categories of Patients. Antimicrob. Agents Chemother..

[22] Dudhani R.V., Turnidge J.D., Coulthard K., Milne R.W., Rayner C.R., Li J., Nation R.L. (2010). Elucidation of the pharmacokinetic/pharmacodynamic determinant of colistin activity against *Pseudomonas aeruginosa* in murine thigh and lung infection models. Antimicrob. Agents Chemother..

[23] Dalfino L., Puntillo F., Mosca A., Monno R., Spada M.L., Coppolecchia S., Miragliotta G., Bruno F., Brienza N. (2012). High-Dose, Extended-Interval Colistin Administration in Critically Ill Patients: Is This the Right Dosing Strategy? A Preliminary Study. Clin. Infect. Dis..

[24] Moni M., Sudhir A.S., Dipu T.S., Mohamed Z., Prabhu B.P., Edathadathil F., Balachandran S., Singh S.K., Prasanna P., Menon V.P. (2020). Clinical efficacy and pharmacokinetics of colistimethate sodium and colistin in critically ill patients in an Indian hospital with high endemic rates of multidrug-resistant Gram-negative bacterial infections: A prospective observational study. Int. J. Infect. Dis..

[25] Trifi A., Abdellatif S., Daly F., Mahjoub K., Nasri R., Oueslati M., Mannai R., Bouzidi M., Ben Lakhal S. (2016). Efficacy and Toxicity of High-Dose Colistin in Multidrug-Resistant Gram-Negative Bacilli Infections: A Comparative Study of a Matched Series. Chemotherapy.

[26] Gibson G.A., Bauer S.R., Neuner E.A., Bass S.N., Lam S.W. (2016). Influence of colistin dose on global cure in patients with bacteremia due to carbapenem-resistant gram-negative bacilli. Antimicrob. Agents Chemother..

[27] Jung S., Chung E.K., Jun M.S., Son E.S., Rhie S.J. (2019). Differences in Colistin Administration and Bacterial and Treatment Outcomes in Critically Ill Patients. Sci. Rep..

[28] Bellos I., Pergialiotis V., Frountzas M., Kontzoglou K., Daskalakis G., Perrea D.N. (2020). Efficacy and safety of colistin loading dose: A meta-analysis. J. Antimicrob. Chemother..

[29] Falagas M.E., Rafailidis P.I., Ioannidou E., Alexiou V.G., Matthaiou D.K., Karageorgopoulos D.E., Kapaskelis A., Nikita D., Michalopoulos A. (2010). Colistin therapy for microbiologically documented multidrug-resistant Gram-negative bacterial infections: A retrospective cohort study of 258 patients. Int. J. Antimicrob. Agents.

[30] Karaiskos I., Giamarellou H. (2014). Multidrug-resistant and extensively drug-resistant Gram-negative pathogens: Current and emerging therapeutic approaches. Expert Opin. Pharmacother..

[31] Pogue J.M., Lee J., Marchaim D., Yee V., Zhao J.J., Chopra T., Lephart P., Kaye K.S. (2011). Incidence of and Risk Factors for Colistin-Associated Nephrotoxicity in a Large Academic Health System. Clin. Infect. Dis..

[32] Katip W., Meechoui M., Thawornwittayakom P., Chinwong D., Oberdorfer P. (2019). Efficacy and Safety of High Loading Dose of Colistin in Multidrug-Resistant *Acinetobacter baumannii*: A Prospective Cohort Study. J. Intensiv. Care Med..

[33] Katip W., Uitrakul S., Oberdorfer P. (2017). Clinical outcomes and nephrotoxicity of colistin loading dose for treatment of extensively drug-resistant *Acinetobacter baumannii* in cancer patients. Infect. Drug. Resist..

[34] Giacobbe D.R., Saffioti C., Losito A.R., Rinaldi M., Aurilio C., Bolla C., Boni S., Borgia G., Carannante N., Cassola G. (2020). Use of colistin in adult patients: A cross-sectional study. J. Glob. Antimicrob. Resist..

